# HIV behavioural interventions targeted towards older adults: a systematic review

**DOI:** 10.1186/1471-2458-14-507

**Published:** 2014-05-26

**Authors:** Joel Negin, Aneuryn Rozea, Alexandra LC Martiniuk

**Affiliations:** 1 Sydney School of Public Health, University of Sydney, Edward Ford Building (A27), Sydney, NSW, Australia; 2 Dalla Lana School of Public Health, University of Toronto, Toronto, Canada; 3 George Institute for Global Health, Sydney, Australia

**Keywords:** Older adults, HIV, Interventions, Systematic review, Effectiveness

## Abstract

**Background:**

The increasing number of people living with HIV aged 50 years and older has been recognised around the world yet non-pharmacologic HIV behavioural and cognitive interventions specifically targeted to older adults are limited. Evidence is needed to guide the response to this affected group.

**Methods:**

We conducted a systematic review of the available published literature in MEDLINE, Embase and the Education Resources Information Center. A search strategy was defined with high sensitivity but low specificity to identify behavioural interventions with outcomes in the areas of treatment adherence, HIV testing uptake, increased HIV knowledge and uptake of prevention measures. Data from relevant articles were extracted into excel.

**Results:**

Twelve articles were identified all of which originated from the Americas. Eight of the interventions were conducted among older adults living with HIV and four for HIV-negative older adults. Five studies included control groups. Of the included studies, four focused on general knowledge of HIV, three emphasised mental health and coping, two focused on reduced sexual risk behaviour, two on physical status and one on referral for care. Only four of the studies were randomised controlled trials and seven – including all of the studies among HIV-negative older adults – did not include controls at all. A few of the studies conducted statistical testing on small samples of 16 or 11 older adults making inference based on the results difficult. The most relevant study demonstrated that using telephone-based interventions can reduce risky sexual behaviour among older adults with control reporting 3.24 times (95% CI 1.79-5.85) as many occasions of unprotected sex at follow-up as participants. Overall however, few of the articles are sufficiently rigorous to suggest broad replication or to be considered representative and applicable in other settings.

**Conclusions:**

More evidence is needed on what interventions work among older adults to support prevention, adherence and testing. More methodological rigourised needed in the studies targeting older adults. Specifically, including control groups in all studies is needed as well as sufficient sample size to allow for statistical testing. Addition of specific bio-marker or validated behavioural or cognitive outcomes would also strengthen the studies.

## Background

HIV prevalence among those aged 50 years of age and older has been rising over the past few years. In the US, in 2005, 25% of those infected with HIV were older than 50 years of age [1] and recent estimates have noted that around 50% of people living with HIV will be older than 50 by 2015 [2]. The number of adults aged 50 years and older living with HIV in the US grew by 14% a year between 2004 and 2007 [3]. Articles have highlighted the HIV and ageing phenomenon in New York [4], London [5], Italy [6] and Australia [7]. The increase has been seen partly due to widespread treatment access [3]. The ageing trend has been increasingly recognised in sub-Saharan Africa where the majority of HIV infections occur [8-11].

The increasing trend of ageing is not only the result of longer survival due to treatment; older adults accounted for 15% of new cases of HIV in the US in 2005 [12]. Western European data reveals that 12.9% of newly reported cases of HIV infection in 2007 were among peopled aged 50 and older compared to 10.4% in 2003 [13]. The percentage of older adults among new infections in Eastern Europe has doubled over the same time frame [13].

The term “older adults” is used to describe those aged 50 years and older. It is acknowledged that in many countries, people in this age group would not be considered old or elderly. In Australia for example, most studies on the elderly focus on those aged 65 years and above or even 70 [14]. The reasons the 50 years cut off point is used here is because the majority of HIV surveillance and reporting over the first two decades of the HIV response has only covered those aged 15–49 [15-17] as well as the accelerated ageing of HIV infected adults compared to uninfected adults [18].

Older adults remain sexually active well into advanced ages and condom use rates among older adults are low [19,20]. In addition, testing rates among older adults are also lower: fewer than 15% of Americans aged over 45 years have been tested for HIV compared to 44% of all adults [21] and late presentation for care is also more common [22]. Older adults – particularly older women – are at greater physiological risk for HIV transmission [23,24]. Those aged 50 and older might also enter into new relationships in which unprotected sex is more likely given the absence of pregnancy concerns [23].

When examined, HIV knowledge amongst older adults has been variable; while some exhibit adequate knowledge many do not consider themselves to be at risk as HIV is considered an illness of younger people [25]. Surveys of HIV positive older women have suggested that insufficient HIV prevention information contributes to risk taking behaviours [26]. Because of this, there have been many urgent calls for prevention messaging and specific HIV programs targeted to older people [23,27-30] rather than simply the application of strategies designed for younger people [31]. This call has extended beyond developed countries to a number of countries in Africa [32,33].

Strengthening the argument for research focussed on HIV positive older adults are the specific problems faced by this cohort [34]. Older HIV positive adults face the additional burden of physical and psychological comorbidities [35] and accelerated senescence [36]. Concurrently, HIV positive older adults experience the double stigma of illness and ageism while having fewer support mechanisms from family, friends and community [37].

Given this situation, a number of academics have lamented the paucity of specific studies conducted among older adults with regard to HIV prevention [38], testing, adherence [39] and other social and behavioural areas [40] for older adults with HIV. There are hypotheses of what would help treatment adherence or the effectiveness of prevention education among older adults including nutrition, mental stimulation, physical activity and better training among physicians [41] but evidence regarding these approaches is patchy and few translated into programmatic use. Orel and colleagues [42] found that in 2004, 15 US States had HIV-related educational materials that were specifically targeted to older adults yet limited evidence is available on their impact on raising awareness and preventing new infections.

The literature provides reviews of the available evidence on HIV behavioural interventions targeted to a range of other affected groups including sex workers [43,44], children [45], men who have sex with men [46], drug users [47] and in occupational settings [48]. Based on these reviews and others, there is mixed evidence on what constitutes effective HIV prevention interventions. Peer education interventions have been found to have some positive impact on risk behaviour in some settings [49] but not others [11]. Routine opt-out testing protocols have demonstrated increased HIV testing uptake in some settings [50]. Internet and text message interventions have shown some positive impact [51,52].

Yet no compilation of evidence exists for older adults despite the recognised trend of an ageing HIV cohort globally. This paper systematically reviews the available published evidence on non-pharmacologic HIV behavioural interventions specifically targeted to older adults with a specific emphasis on treatment adherence, HIV testing uptake, increased HIV knowledge, reduced risk behaviour as well as social and physical support.

## Methods

The systematic review adheres to the PRISMA guidelines. Searches were conducted on 6 February 2012 and again on 4 June 2012 in MEDLINE, Embase and the Education Resources Information Center (ERIC). A search strategy was defined with high sensitivity but low specificity. The search strategy consisted of free-text and Medical Subject Headings (MeSH) terms. Search terms for older adults included the following: “elderly”, “older adults”, “aged”, “ageing”, “geriatric”, “mature adults” and “senior citizen”. The terms HIV, AIDS and HIV/AIDS were included. Additional search terms to identify interventions or trials included “intervention”, “trial”, “evaluation”, “intervention studies”, “randomized controlled trials”, “evaluation studies”, “program evaluation” and “prevention”, “testing” and “adherence” to ensure capture of relevant articles. Between each of the three categories of terms, an “AND” term was included so that only articles including information on HIV and older adults and relevant interventions were included in the results. Additionally, references of identified publications and published reviews were hand searched for potential additional relevant articles.

There were no restrictions on language or year of publication or country. We included studies that reported specifically on those aged 50 years and older or that reported age groups in which the majority of respondents were in that age group (i.e. 45+). Studies that included participants aged, for example, 25–52 were excluded if there was no specific sub-group reporting on those aged 50 and older or in which a majority of respondents were aged 50 and older.

**Figure 1:**
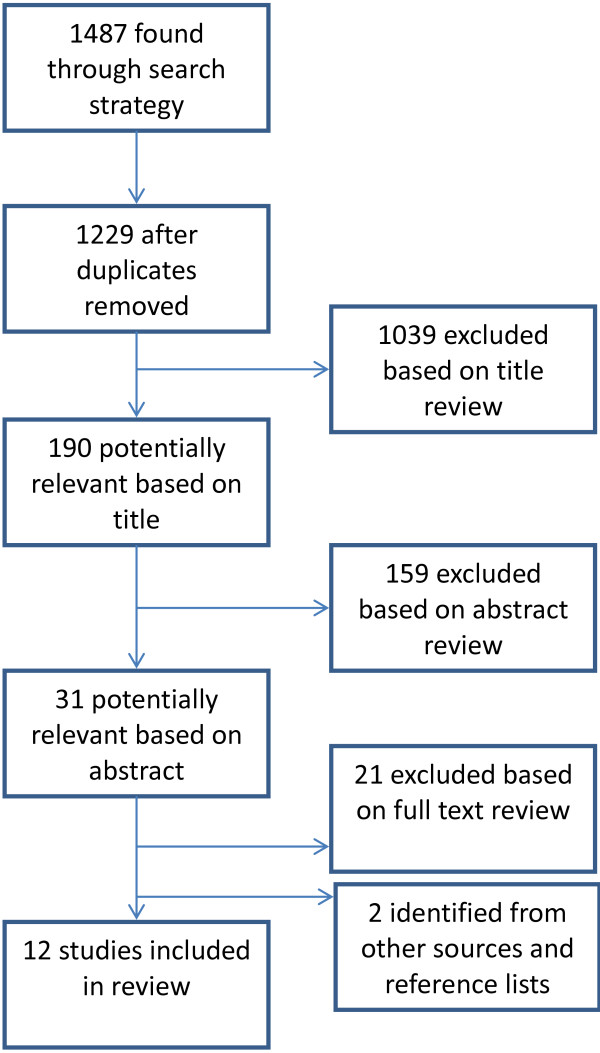
Selection of manuscripts for systematic review of behavioural HIV interventions targeted to older adults.

The inclusion criterion of the review was the search for non-pharmacologic, non-biological, behavioural and cognitive interventions. We excluded studies that evaluated treatment efficacy among older adults and included those that focused on interventions in the areas of prevention, adherence, testing, care and support. We were particularly interested in studies that demonstrated outcomes in treatment adherence, HIV testing uptake, increased HIV knowledge, reduced risk behaviour (related to sexual behaviour or injecting drug use in particular) as well as social and physical support.In the first review round, two reviewers (JN, AR) independently scrutinised the list of article titles and eliminated the clearly non-relevant citations. Another round of exclusions using abstracts of those articles deemed potentially relevant based on titles was conducted and the final selection was based on the full text of potentially relevant articles. In cases of disagreement, a third reviewer (AM) examined the articles. Results and inclusion were discussed until consensus was reached among all three reviewers. Figure 1 outlines the number of studies included at each stage of the review process.

Data were then extracted from each relevant study into an excel spreadsheet. Extracted information included year, location of study, brief description, study type, sample size, study population, review of methods, primary outcome measure and focus topic of the study. Major themes were identified in the relevant studies. The study adheres to the PRISMA guidelines for systematic reviews.

## Results

After removing duplicates, the search revealed 1229 articles. After a review of titles and then abstracts, ten articles met study criteria. An addition two articles were identified through searching reference lists and were included in the review providing twelve articles in total.

All twelve of the included studies originated from the Americas: two from Brazil and the rest from the United States. Eight of the interventions were conducted among older adults living with HIV and four among HIV-negative older adults (Table 1). Five studies included control groups.

**Table 1:** Summary of included articles

**Article**	**Focus area**	**Participants**	**Study design**	**Intervention**	**Control group (Y/N)**	**Measure**	**Results**	**Summary**
Among HIV-Negative Older Adults								
Small 2009 [60]	Knowledge	Individuals aged 50 years and older N = 50	Both quantitative and qualitative data were gathered during each session and included a pre-survey and immediate post-37-item-survey, a focus group, and an HIV educational curriculum.	Four HIV education training sessions each lasting three hours covering the following topics: (a) Introduction and Overview, (b) Identifying Myths and Stereotypes, (c) HIV Facts, and (d) Provision of Resources.	No	Interest in HIV prevention and education (composite of two questions)	HIV knowledge was not significantly (p = .273) higher in the posttest (mean = 10.92) than in the pretest (mean = 10.32)	Substantive knowledge about HIV and AIDS remained low among respondents, and there was no significant change in knowledge after the administration of the modified curriculum
		Purposive sampling USA				Substantive HIV knowledge (composite of 15 questions)		
Orel et al. 2010 [58]	Knowledge	11 participants of 89 aged 60 and older completed both questionnaires N = 11 USA	Before and after questionnaires	“No One is Immune Project” –six-hour education and prevention workshop held at senior centre. Post-test questionnaire administered immediately after workshop.	No	45-item HIV/AIDS Knowledge Questionnaire	Increase in % answering correct	Workshop increased knowledge of HIV among older adults
							● HIV can be spread by mosquitoes (48 to 98)	
							● you cannot get HIV when getting a tattoo (25 to 78)	
							● women are always tested for HIV during their pap smears (12 to 100)	
							● No data provided on overall changes in scores	
Altschuler et al. 2004 [59]	Knowledge	Adults aged 50 years and older USA N = 40	Verbal feedback after educational program	3 hour educational program. Curriculum includes overview, myths and stereotypes, facts and resources.	No	Group feedback on HIV awareness, HIV perceptions and ability to speak to health care providers	Participants identified learning that HIV was relevant to their lives; feeling empowered to speak up to their health care providers; positive impression being able to discuss a taboo topic.	Qualitative results suggest that education program can help individuals discuss HIV with partners.
Rose 1996 [25]	Knowledge	Individuals aged >60 recruited at senior citizen meal sites USA	Pre and post-test cross-sectional survey	20-30 minute age-specific AIDS education program delivered at meal site and educational pamphlet. Consisted of statistics and facts about HIV, prevention measures and case studies of elderly people with HIV. Post-test administered immediately following education program.	No	Questionnaire measured HIV knowledge and perceptions of susceptibility to HIV (Likert scales)	Significant increase in total knowledge about AIDS (p < 0.001), perceived susceptibility (p < 0.01) and perceived severity (p < 0.001)	Age specific education program significantly increased HIV-related knowledge at senior citizen meal sites
		Pre-intervention N = 458						
		Post-intervention N = 318						
Among HIV-Positive Older Adults								
Lovejoy et al. 2011 [53]	Risk behaviour	HIV-infected adults 45-plus years old who reported engaging in at least one occasion of unprotected sex in the 3 months prior to enrolment N = 100 USA	Randomised controlled trial	Telephone delivered motivational interviewing (MI) (client-focused and directive form of counselling) to reduce risky sexual behaviour	Yes (N = 23)	Episodes of unprotected sex in past 3 months	Participants in the 4-session MI arm engaged in the fewest occasions of unprotected sex at 3 and 6 month follow-up. Controls had on average 3.24 times as many occasions of unprotected sex (95% CI 1.79-5.85). Furthermore, 1-session MI participants had four times as many unprotected sex acts as 4-session MI participants at 3-month (OR = 3.98 [2.38–6.67]) and 6-month (OR = 4.39 [2.56–7.46]) follow-up.	Four sessions of telephone-delivered MI reduces sexual risk behaviour among HIV-positive older adults
			3 arms – 4-sessions (N = 39), 1 session (N = 38) or nothing/control (N = 23).					
Ruiz and Kamerman 2010 [57]	Referral for care	HIV-positive patients aged >60 years N = 57 USA	Descriptive	Functional screening for detection of comorbidities and referral for further care if failed in 3 or more domains	No	Referrals for comorbid conditions	17 patients were referred due to problems in multiple domains including cognitive dysfunction (10), problems in daily living (8), nutritional issues (6), depression (5), and mobility (5)	Screening for comorbidities among HIV-positive older adults can facilitate referral for further care likely to improve quality of care and outcomes
Illa et al. 2010 [54]	Risk behaviour	HIV-positive, 45 or older, sexually active in last 12 months	Randomised controlled trial	Project ROADMAP (re-educating older adult in maintaining AIDS prevention).	Yes	Sexual risk (number of partners, partner HIV status, sexual acts, condom use)	Inconsistent condom use with partners of negative or unknown serostatus reduced from 9% at baseline to 1.3% at 6 month follow-up among intervention group (p = .003); reduced from 4% to 3% with control group (p = .999)	Group psycho-educational sessions reduced unprotected sexual acts with partners of unknown or negative serostatus
				Intervention group: educational brochure and four psycho-educational group sessions designed for HIV-positive older adults. Sessions focused on information, motivation, behavioural skills and risk reduction behaviours. Control: educational brochure only.				
		N = 241 (149 intervention group and 92 in control group) USA						
						HIV knowledge (33 item measure) Sexual self-efficacy		
Heckman et al. 2001 [37]	Coping	HIV-positive individuals aged 50 years and older attending AIDS service organizations	Pilot pre- and post-test cohort study	Coping improvement group intervention	No	Severity of HIV-related Life Problem Scale	Increased social wellbeing (2.20 to 2.41, p < 0.05)	Group sessions focusing on coping strategies had limited impact on coping and stress but increased social wellbeing among HIV-positive older adults
						Ways of coping Questionnaire Functional assessment of HIV Infection Scale		
							Marginal non-significant change in coping (p < 0.10)	
							Marginal non-significant decrease in stress associated with AIDS-related loss and health concerns (p <0.10)	
						Provision of Social relations		
							Marginal non-significant increase in support from friends (p < 0.10)	
Heckman et al. 2006 [55]	Coping	HIV-positive individuals aged 50 years and older recruited from AIDS service organizations with diagnosis of depression or dysthymia N = 90 USA	Randomised controlled trial with delayed treatment control Embedded cohort study Assessed pre, post (within 1 week of completion) and at3 months	12, 90 minute sessions, telephone delivered weekly coping improvement group intervention to reduce psychological distress 1) Immediate treatment (n = 44) 2) Delayed treatment (control) (n = 46)	Yes, delayed treatment, control group	Geriatric depression scale (GDS) Symptom checklist 90-revised (SCL-90-R)	Intervention group reported fewer psychological symptoms (p = 0.05), less life stressor burden (p = 0.058), less use of avoidant coping strategies (p = 0.05) and marginally higher levels of coping self-efficacy (p = 0.10) compared to controls with no effect on depressive symptoms, loneliness or use of engagement coping **Within cohorts IG:** significant decrease in depressive symptoms (p < 0.003) psychological symptoms (p < 0.001), life-stressor burden (p < 0.03) and avoidance coping (p < 0.04) at 3 months Delayed group: significant decrease in psychological symptoms (p < 0.03), life stressor burdens (p < 0.001), loneliness (p < 0.03) and greater coping self-efficacy (p < 0.04) following intervention	Telephone delivered coping group sessions among HIV-positive people with depression were effective in reducing psychological symptoms and stress
						HIV-related life-stressor burden scale UCLA Loneliness Scale (10-item)		
						The Ways of Coping Checklist (WOCC)		
						Coping self-efficacy Scale		
Heckman et al. 2011 [35]	Coping	HIV-positive individuals aged 50 years or older with Beck Depression Inventory-II score 10 or more and Modified mini-mental state examination score of 75 or greater. N = 295 USA	Randomised controlled trial 3 arms	1) 12 90 minute sessions face-to-face coping improvement (FFCI) group intervention (n = 104)	Yes	Geriatric Depression Screening Scale	Both FFCI and IPSG participants reported fewer depressive symptoms than controls post-intervention, 4- and 8-month follow-up. This effect was not always statistically significant (p’s < 0.01-0.1). IPSG reported fewer depressive symptoms compared to control.	An age-appropriate coping improvement group intervention was effective in reducing depressive symptoms in HIV + older adults. The effect was more pronounced amongst subjects suffering greater levels of depressive symptoms.
						Completed using audio-computer assisted self interviews (A-CASI)		
				2) 12 session interpersonal support group (IPSG) intervention (n = 105)				
				3) Individual therapy upon request (ITUR) control (n = 86). Subjects had access to standard psychosocial services available in the community.				
							Effect size greater for subset of participants with mild, mod and severe depression at baseline	
Souza et al. 2008 [56]	Physical support	Subjects HIV+, older than 60 (Mean 65.6+/- 2.9), sedentary at baseline. 3 subsequently excluded due to >3/12 absence from training program N = 11.	Prospective Case series study	1 year resistance training program 4 exercises targeting major muscle groups 3 sets 8-12 reps at light, mod and heavy resistance respectively 2 sessions/week, one year	No	***Anthropometric indices:*** Body mass, circumferences and skin folds Body composition (DEXA) ***Strength and functional tests:*** Sub-maximum weight lifted Two functional tests performed every 4 months assessing walking speed, and sit-to-stand performance.	No significant change in weight Strength improvements of between	Following one year of progressive resistance training HIV positive older adults showed significant improvements in strength and functional capacity, no changes in body composition and improved immunological indices.
							74-97% (p’s = 0.003-0.021)	
							Functional tests: decreased times for sit-stand (2.00 to 1.57 s, p = 0.003) and walking 2.4 m (9.25 to 6.58 s, p = 0.003)	
		All subjects medical able to complete training and not using cortico- or anabolic steroids Brazil						
Souza et al. 2011 [36]	Physical support	Subjects HIV+, age > = 60 (M = 64.4 +/- 3.0) Ave. 9 year history of HIV, recruited at Hospital in Sao Paulo, Brazil N = 11 with 21 controls All subjects medically able to complete training and not using cortico- or anabolic steroids	Controlled trial	Progressive resistance exercise 2 sessions per week for 1 year.	Yes, age, activity and gender matched HIV–controls (N = 21)	***Strength and functional tests:*** Sub-maximum load monitored bi-monthly Functional test of walk and sit-stand speeds. ***Anthropometric and metabolic indices:*** Weight; BMI Lipid and glycaemic profiles (values registered in clinical record immediately before and after training program)	Although weaker at baseline, HIV + subjects increased weight lifted from 1.52 to 2.33 times baseline, a significantly greater improvement than controls (1.21-1.48, p < 0.01) HIV + lighter, significantly lower BMI (p = 0.007 pre and p = 0.004 post) Faster at walking tests (significant, p = 0.036 pre-, not significant post-training). HIV + significantly faster at sit-to-stand after training than controls (p = 0.005) Fasting BSL significantly improved in both groups (p’s = 0.027-0.037).	Progressive resistance exercise training produced increased strength and functional gains in older adults living with HIV, with gains superior to those seen in age-matched HIV- controls. An effect was also seen on metabolic indices.
				Five exercises utilised major muscle groups				
				3 sets 12/10/8 repetitions at sub-maximum load				

Of the included studies, none were focused on improving treatment adherence and none had increased HIV testing uptake as an outcome measure. The four articles conducted among HIV-negative participants aimed to improve general HIV knowledge to facilitate HIV prevention. Within the studies among HIV-positive participants, the main outcome measure of Lovejoy and colleagues [53] and Illa and colleagues [54] articles was reduced sexual risk behavior. The three Heckman et al. articles [35,37,55] focused on mental health and coping assessments while the two Souza et al. papers [36,56] emphasized physical status and strength. Improved referral for care was the outcome measure of the last relevant article [57].

The included studies do highlight areas for action in developing prevention and adherence interventions specifically for older adults. With respect to improving knowledge, Rose demonstrated that delivery of an age-specific AIDS education program was effective in improving HIV knowledge (p < 0.001) and perceptions of susceptibility (p < 0.01) among older HIV-negative Americans compared to a control group not receiving the education program [25]. While Orel and colleagues [58] and Altschuler and colleagues [59] both showed that education sessions improved HIV-related knowledge among older adults, Small’s workshop did not have a significant impact [60].

Other interventions targeted HIV-infected older adults to reduce risk behaviour. The ROADMAP (Reeducating Older Adult in Maintaining AIDS Prevention) project ran group educational sessions to reduce high-risk sexual behaviours [54]. After sessions that included practicing condom use skills and role playing condom negotiation, participants in the intervention group (N = 149) reported reduced inconsistent condom use (9% at baseline to 1% at 6 month follow-up; p = 0.003) compared with the control group (N = 92) (4% at baseline to 3% at follow-up; p = .0999). Another study used telephone-based interventions to reduce risky sexual behaviour with controls reporting on average 3.24 times (95% CI 1.79-5.85) as many occasions of unprotected sex at follow-up [53].

Supporting older adults to cope with HIV infection was the focus of three papers [35,37,55]. Heckman and colleagues reported that group sessions with trained facilitators that included a focus on coping mechanisms and how to deal with stress are effective in reducing depressive symptoms in HIV-positive older adults (p values ranging from 0.01 to 0.1 for variety of measures; see Table 1) [35].

Weekly resistance exercise training sessions trialed in Sao Paulo, Brazil [36,56], among HIV-positive older adults, produced increased strength and functional gains superior to those seen in age-matched HIV-negative controls. Those receiving the intervention (N = 11) increased weight lifted compared to controls (N = 21) (p < 0.01) and were faster at sit-to-stand after training (p = 0.005) [36].

## Discussion and conclusion

This systematic review of behavioural HIV interventions specifically targeted to older adults reveals the absolute paucity of published literature on this topic despite the increases in the number of people living with HIV aged 50 years and older and the increasing prevalence in this group globally. Though there have been many calls for more evidence that specifically address the vulnerabilities and characteristics of older adults, only twelve articles were found that included some evaluation of an intervention for this population. Limited conclusions can be drawn due to the small sample of relevant studies. The diversity of behavioural and cognitive interventions applied in the studies limits the ability to generalise from the results available; a number of interventions focused on knowledge building, others on sexual behaviour and others on coping.

Only four of the studies were randomised controlled trials and seven – including all of the studies among HIV-negative older adults – did not include controls at all. A few of the studies conducted statistical testing on small samples of 16 or 11 older adults making inference based on the results difficult. A number of the studies – especially those among HIV-negative individuals focused on general HIV awareness and interest as opposed to behaviour change. More methodological rigour is needed in the studies targeting older adults. Specifically, including control groups in all studies is needed as well as sufficient sample size to allow for statistical testing. Addition of specific bio-marker or validated behavioural or cognitive outcomes would also strengthen the studies.

The majority of articles identified from the searches and ultimately excluded from this review describe behaviours of older adults rather than evaluating an intervention [34]. For example, there were articles that described specific intervention projects targeted to older adults – for example the Florida Senior HIV Intervention Project – but which did not provide any evidence of success or evaluation of the program [61]. Other papers did not report on those aged 50 and older. For example, one paper provided data for the age group 30–59 but was not included in the review because it did not report on older adults specifically [62].

Despite the limited number of papers providing evidence on this topic, some lessons do emerge from the review. Due to concerns over stigma and the perception of being low risk, researchers have suggested approaches for older adults through the lens of sexuality and sexual health rather than HIV directly [58]. Providing information on, for example, the impact of medications on sexuality may provide an appropriate time to discuss safe sex and HIV.

Though there is an erroneous ageist assumption that older people are not able to make behaviour changes [63], a number of ideas were expressed as possible avenues to reach older adults with HIV services including using older adults as peer educators [42] and providing training in sexuality counselling to social workers working with this age group [40]. Others advocate for the increased use of internet and mobile phone based HIV programs to reach the elderly who might be uncomfortable discussing HIV in more public settings [64]. A number of authors called for more education of physicians on the topic to ensure that HIV counselling and also testing are carried out among older adults [65,66].

This systematic review has a number of limitations. The review focused specifically on interventions reporting outcomes among older adults and therefore might have missed interventions designed for adults of all ages that are effective among older adults. The review only included published papers documenting interventions that had been evaluated thus eliminating a large set of publications that only describe interventions. The review is also subject to publication bias and selective reporting as is common in systematic reviews.

The review only searched for studies specifically on HIV. There are a number of other studies on medication adherence generally among older adults [67-69] or on sexual health interventions that might be relevant to HIV that were not included here.

Given the trends within the HIV community, much more rigorous evidence is needed on how best to provide services for those aged 50 years and older. A number of interventions were mentioned in articles, but without evidence of impact, suggesting that there are opportunities for evaluations to add to the base of knowledge in this area [61]. Some particular understudied challenges for older adults include: 1) through which channels is it best to reach older adults; 2) what types of messaging are most appropriate for this population; and 3) what content resonates with older adults. Interventions might be targeted to older adults themselves or to health professionals and other providers of services to older adults. Specific evaluated interventions are needed in the areas of testing, supporting adherence, reducing risky sexual behaviours are all areas where the evidence base is currently insufficient. More operational research is needed to scrutinise the “how” of service delivery for older adults [70] and how to provide it at scale. The other major research challenge will be building an evidence base for older adults living in low and middle income countries where very little currently exists. The American studies are likely not to be applicable to other parts of the world and therefore new research will be critical as the HIV cohort in Africa and Asia ages into the future.

## Competing interests

The authors declare that they have no competing interests.

## Authors’ contributions

JN conceived of the study. JN and AR conducted the initial reviews of articles and AM served as arbitrator. JN and AR wrote the first draft and AM provided reviews. All authors read and approved the final manuscript.

## Pre-publication history

The pre-publication history for this paper can be accessed here:

http://www.biomedcentral.com/1471-2458/14/507/prepub
